# A166 RISK STRATIFICATION OF EARLY RE-HOSPITALIZATION IN PERSONS WITH INFLAMMATORY BOWEL DISEASES USING MULTIVARIABLE MODELS

**DOI:** 10.1093/jcag/gwac036.166

**Published:** 2023-03-07

**Authors:** C Dziegielewski, S Gupta, J Lombardi, E Kelly, J McCurdy, R Sy, T Ramsay, J Begum, S Murthy

**Affiliations:** 1 Department of Medicine, University of Ottawa, Ottawa; 2 Department of Medicine, University of Toronto, Toronto; 3 Department of Anesthesia, McMaster University, Hamilton; 4 Ottawa Hospital Research Institute; 5 The Ottawa Hospital; 6 University of Ottawa; 7 ICES uOttawa, Ottawa, Canada

## Abstract

**Background:**

Hospitalization for persons with inflammatory bowel disease (IBD), including Crohn’s disease (CD) and ulcerative colitis (UC), is a significant contributor to morbidity and health care costs in Canada. Recognition of individuals at high risk of re-hospitalization could help inform targeted outpatient interventions that mitigate this risk.

**Purpose:**

The aim of our study is to derive prediction models of risk of early (90-day) re-hospitalization among persons with IBD.

**Method:**

We conducted a retrospective cohort study of all adult persons with IBD admitted to The Ottawa Hospital, Canada, for an acute IBD-related indication between April 2009 - March 2016. Demographic, clinical, and health services variables were obtained through chart review. Persons were linked to population-based health administrative datasets to identify historical and future IBD-related hospitalizations across the greater Ottawa region. Multivariable logistic regression models of 90-day re-hospitalization in persons with CD and UC were derived, and candidate predictors that demonstrated an independent association with the outcome at a p-value of 0.1 were retained. Bootstrap internal validation (200 iterations) was performed on the final models. Model performance and calibration were evaluated using the optimism-corrected c-statistic value and Hosmer-Lemeshow goodness of fit test, respectively. Adjusted odds ratios are reported with 95% confidence intervals (CI). Optimal probability cut points for re-hospitalization were selected to optimize sensitivity, specificity, and the J (Youden’s) index.

**Result(s):**

There were 524 CD and 248 UC hospitalizations during the study period. Of these, 57 (10.9%) CD and 27 (10.9%) UC hospitalizations were associated with re-hospitalization within 90 days of discharge. Forty-two candidate predictors were tested among CD hospitalizations, and 35 were tested among UC hospitalizations. Four variables were retained in each of the final models. Model performance and calibration for each variable are described in Table 1. The optimal range of probability cut points allowed for a sensitivity/positive predictive value (PPV)/false positive rate (FPR) of 0.72/0.23/0.29 (maximum J-index of 0.43) in the model for CD, and 0.78/0.33/0.19 (maximum J-index of 0.59) in the model for UC, respectively.

**Image:**

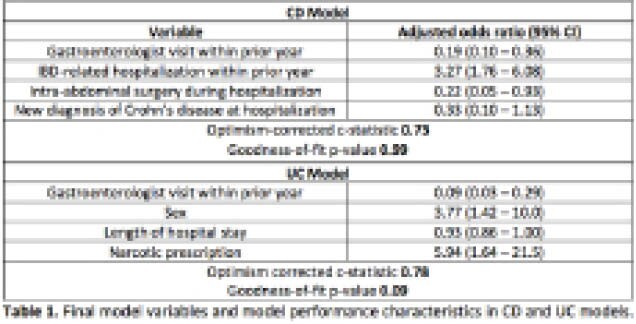

**Conclusion(s):**

Demographic, clinical, and health services variables at the time of discharge have the potential to help identify persons with IBD at risk of early re-hospitalization, thereby permitting targeted outpatient intervention. Application of the models to our reference cohorts would earmark 1/3 or less of patients for early post-discharge intervention, with the potential to benefit more than 70% of patients destined for early re-hospitalization. Although the PPVs of our models were low, the models incorrectly predicted early re-hospitalization in less than 30% of patients. We are in process of externally validating these models in other jurisdictions across Ontario to test their generalizability.

**Please acknowledge all funding agencies by checking the applicable boxes below:**

None

**Disclosure of Interest:**

None Declared

